# Covalent Attachment of Molecularly Thin PVC Membrane by Click Chemistry for Ionophore-Based Ion Sensors

**DOI:** 10.1021/acs.analchem.5c01986

**Published:** 2025-08-11

**Authors:** Yupu Zhang, Tara Forrest, Yaotian Wu, Plinio Maroni, Eric Bakker

**Affiliations:** Department of Inorganic and Analytical Chemistry, University of Geneva, Quai Ernest-Ansermet 30, CH-1211 Geneva, Switzerland

## Abstract

Solid-contact ion-selective electrodes (SC-ISEs) are widely used in clinical diagnostics devices and are expanding into other fields including environmental and wearable sensors. Despite significant materials research efforts, the most widely used polymer for membrane preparation remains plasticized poly­(vinyl chloride) (PVC). Owing to its nonreactive nature, it is normally applied onto the electrode substrate by evaporative solvent casting. Unfortunately, the resulting films tend to adhere poorly onto the electrode substrate and it is difficult to fabricate robust, well-defined sensing films with controlled thickness down to the nanoscale. This work introduces, for the first time, a PVC polymer membrane that is chemically bonded to the electrode substrate by Cu­(I)-catalyzed azide–alkyne cycloaddition (click chemistry). A molecularly thin PVC membrane in which a fraction of the chlorine atoms of the PVC are replaced by azide functionalities is covalently attached to an electropolymerized PEDOT substrate containing alkyne groups by click chemistry. Electrochemical quartz crystal microbalance studies during the electropolymerization and membrane click reaction procedures suggest layer thicknesses of 776 and 42 nm of the conducting polymer and PVC membrane layer, respectively. Once doped with plasticizer and ion sensing components, the ultrathin PVC membrane exhibited a near-Nernstian response slope and excellent ion selectivity. Analytical properties were further improved by overcoating the PVC film with solvent cast PVC, resulting in a standard deviation of the *E* ^0^ value for potassium-selective electrodes of 1.60 mV.

## Graphical abstract

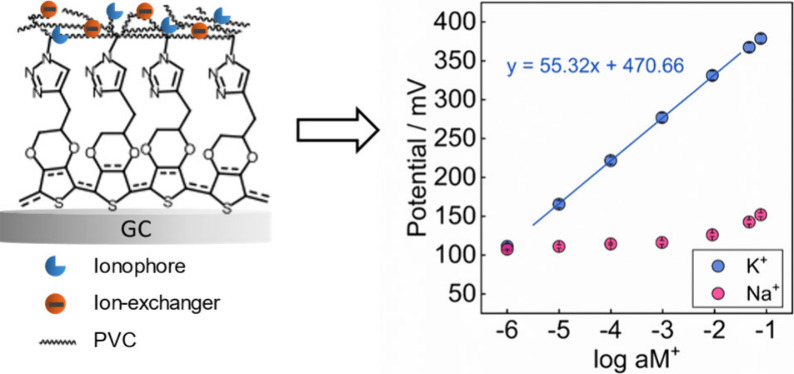

In recent decades, solid-contact ion-selective electrodes (SC-ISEs) have been developed and applied in many fields, owing to their adequate stability, high ion selectivity, small size, and low cost.^1–4^ Ion-selective membranes (ISMs) are prepared using evaporative solvent casting, resulting in membranes with thicknesses of tens or hundreds of micrometers. This ensures good sensor performance and stability but necessitates relatively long preparation and conditioning times. To address this, some studies have reduced ISM thickness further. For example, a 200-nm-thick membrane was obtained by spin-coating onto a GC electrode to allow for a selective ion response and multi-ion detection via ion-transfer voltammetry.^5,6^ Unfortunately, such ultrathin membranes exhibit limited longevity and mechanical robustness. Moreover, PVC is known to adhere poorly onto most substrate materials and there is an important need for well-controlled, ultrathin polymer membranes exhibiting strong adhesion.

As the quintessential click chemistry reaction, Cu­(I)-catalyzed azide–alkyne cycloaddition (CuAAC) was independently introduced by K. Barry Sharpless and Morten Meldal.^7,8^ For ISEs, click chemistry has been used to modify substrate materials and conducting polymers, achieving improved stability and enhanced performance.^9–11^ Our group has previously developed a novel PEDOT transducing layer functionalized with lipophilic plasticizer-like side chains via CuAAC to improve the hydrophobicity of the ion-to-electron transducing layer.^9^

We propose here, for the first time, a novel strategy to chemically bond azide-modified PVC onto a conducting polymer substrate containing alkyne groups. This results in a molecularly thin ion-selective membrane that cannot readily be lost by desorption or delamination. This molecular architecture should provide an improved control of the materials properties and thickness of such membranes. Specifically, as shown in Figure 1, a conducting polymer, PEDOT-alkyne, is electrodeposited from 2-pent-4-ynyl-2,3-dihydro-thieno­[3,4-*b*]­[1,4]­dioxine (EDOT-alkyne) through two voltammetric cycles (Figure S1 and the Experimental Section in the Supporting Information). Subsequently, a molecularly thin PVC membrane is covalently attached through CuAAC between PVC-N_3_ and PEDOT-alkyne.^12^

**Figure 1:**
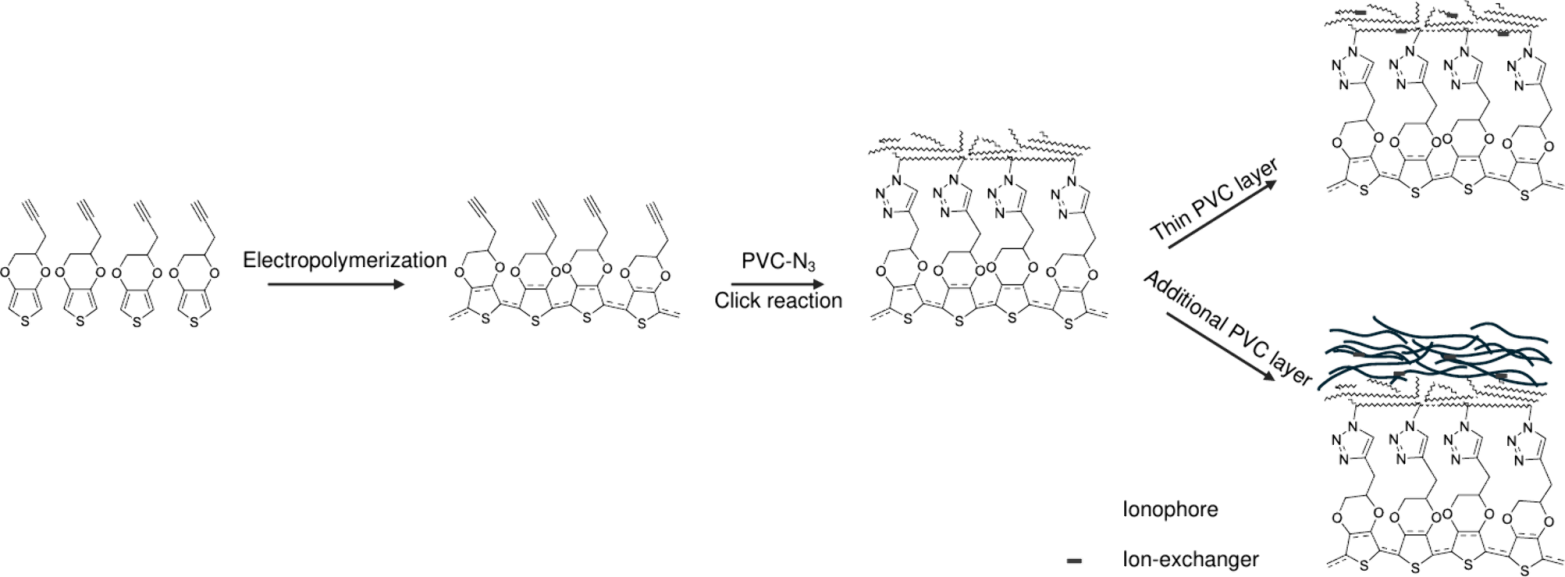
Schematic of the fabrication process for PVC-attached ion-selective electrodes, featuring both a molecularly thin PVC layer and a thick PVC layer (with an additional PVC membrane).

Two strategies are employed to incorporate other sensing components. The first involves directly doping the ionophore, ion exchanger, and plasticizer into the covalently attached PVC membrane (Figure 1, top-right). The second overcoats a cocktail containing additional PVC along with other components, forming an additional PVC layer on the clicked PVC membrane (Figure 1, bottom-right).

Electrochemical quartz crystal microbalance (QCM) experiments were performed to monitor the mass change during the reaction process (Figure 2). The PEDOT layer is formed through two cycles of electropolymerization, and two distinct peaks are observed when the potential exceeds about 1 V during the deposition process, which is thought to correspond to the two polymerization cycles.^13^ The mass change during click reaction was monitored (see Figure 2b). A noticeable mass increase for the substrate was observed after the addition of catalyst. After about 86 min, the mass was found to stabilize, indicating the completion of the click reaction. This suggests that such click reactions between azide and alkyne are complete within 2 h.

**Figure 2:**
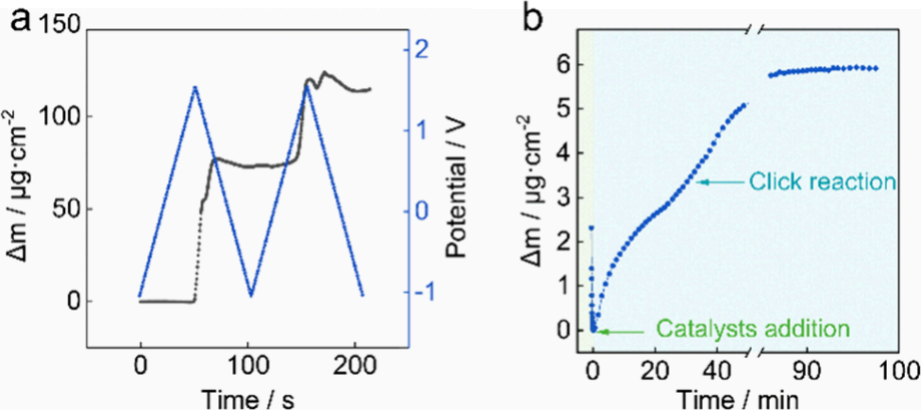
Mass change monitored by electrochemical quartz crystal microbalance during (a) EDOT-alkyne electropolymerization process (black scatters: mass change, blue scatters: applied potentials) and (b) click reaction between PVC-N_3_ and PEDOT-alkyne.

Based on the mass difference (113.97 ± 0.01 μg·cm^–2^) and an estimated density of PEDOT-alkyne, the layer thickness was calculated as about 775.31 ± 0.01 nm. As no specific reference for the density of PEDOT-alkyne is available, the density of PEDOT (1.47 g·cm^–3^) was used as approximation.^14^ A similar estimation method was applied to determine the thickness of the attached PVC membrane, which was calculated as 42.97 ± 0.01 nm (5.93 ± 0.01 μg·cm^–2^ mass difference, 1.38 g·cm^–3^ density), demonstrating an 18-fold thinner membrane film than the conducting layer.^15^ The possible thickness of the clicked PVC membrane may vary significantly. In one limiting case, most azide groups of the PVC would react with the alkyne groups on PEDOT layer, resulting in PVC chains horizontally arranged on the conducting polymer surface, where the thickness of the PVC layer should correspond to the distance between several parallel PVC molecules, measuring only a few nanometers.^16^

In the second limiting case, only a small fraction of azide groups react with the alkyne groups on the substrate. This should cause PVC molecules to adopt a vertical, densely packed arrangement with a thickness approaching the length of a single PVC polymer chain. With high-molecular-weight PVC (233 000 Da), an average polymer length of about 700 nm may be estimated. Based on the thickness obtained from QCM (42.8 nm), it may be inferred that the PVC chains are neither perfectly horizontally arranged nor fully vertically aligned but instead form folded chains and overlapping structures. Despite this, the PVC membrane remains ultrathin, which may offer several potential advantages including faster equilibration times, simpler preparation, and potentially enhanced sensitivity while remaining covalently bonded to the substrate. It was attempted to characterize the thickness of PEDOT layers by ellipsometry with a specific layer model as described in the Experimental Section. Unfortunately, no thickness information could be obtained from the experimental data. One possible explanation is the strong absorption of the PEDOT layer, which leads to weak measurement signals and imprecise estimations.

To evaluate the degree of completion for the click reaction, cyclic voltammetry (CV), water contact angle (WCA) measurements, and electrochemical impedance spectroscopy (EIS) were performed (Figure 3). The redox peaks of PEDOT-alkyne were no longer visible after the attachment of PVC after the click reaction, which is attributed to the reduced conductivity of PVC (Figure 3a). The water contact angle showed a slight increase from 108.0° to 112.0° (see Figure 3b, as well as Figure S2). This modest change is understandable, given the inherently hydrophobic nature of the PEDOT layer.^17^ EIS further confirmed the attachment of PVC (Figure 3c), with the corresponding equivalent electrical circuits shown in Figure S3. Before the click reaction, the system exhibited a typical Randles circuit with a charge transfer resistance of 142.00 Ω. Following the click reaction, the presence of a semicircle in the midfrequency range indicated an additional resistance (film resistance), confirming successful PVC attachment.

**Figure 3:**
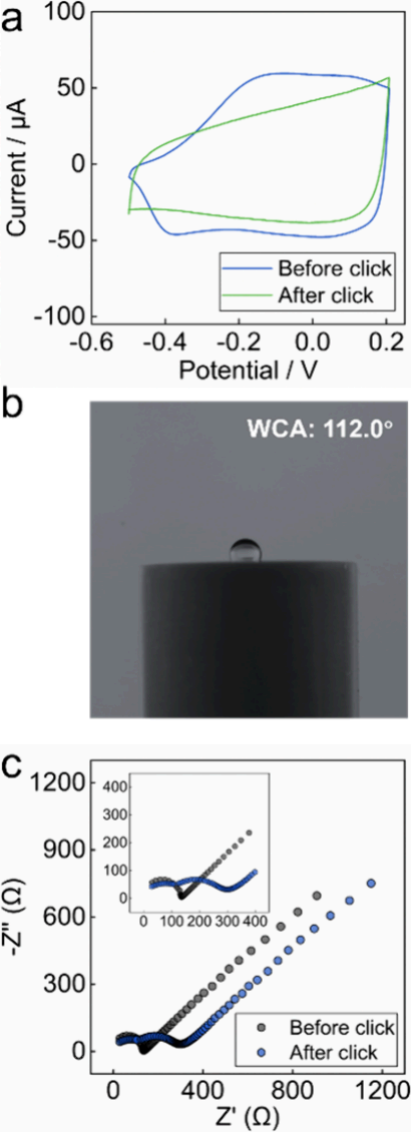
(a) Cyclic voltammograms of a GC electrode with a PEDOT-alkyne layer before and after the click reaction (supporting electrolyte, 0.1 M TBAClO_4_; scan rate, 100 mV/s). (b) Water droplet image after the click reaction. (c) Nyquist plots for the electrode before and after the click reaction in a solution with 0.1 M KCl and 0.01 M K_3_[Fe­(CN)_6_]/K_4_[Fe­(CN)_6_]. Impedance measurements were taken using an amplitude of ± 10 mV rms over a frequency range of 0.01–10^5^ Hz.

Based on this molecularly thin membrane substrate, we directly added a cocktail containing potassium ionophore, ion exchanger, and plasticizer (without additional PVC). A possible concern is whether a nanometer-thin PVC membrane can maintain electroneutrality and provide sufficient Donnan exclusion to achieve Nernstian slopes, given that the membrane thickness is less than 1 μm. Morf et al. discussed similar cases involving a 30-nm membrane containing fixed anionic sites and exchangeable cations in an asymmetric configuration.^18^ In that work, the membrane is not strictly electroneutral but can still exhibit a near-Nernstian response, albeit with reduced long-term stability and weakened Donnan exclusion.^18^ Moreover, ion-selective electrodes based on other types of ultrathin membranes, such as bilayer lipid membranes that are typically only a few nanometers thick, have demonstrated Nernstian behavior experimentally.^19^

As shown in Figure 4, the covalently attached molecularly thin PVC membrane electrode indeed demonstrated a Nernstian response of 55.3 ± 0.5 mV (same electrode measured repeatedly) for K^+^ with excellent Na^+^ selectivity (log *K*_*I*,*j*_^pot^ = – 4.10). Figure S4 shows that, in some cases, sub-Nernstian response slopes are observed, which are attributed to membrane thickness inhomogeneity that may result in Donnan failure. The data indicate that a molecularly thin PVC membrane is capable of adequately incorporating sensing components and achieving attractive ion sensing performance.

**Figure 4:**
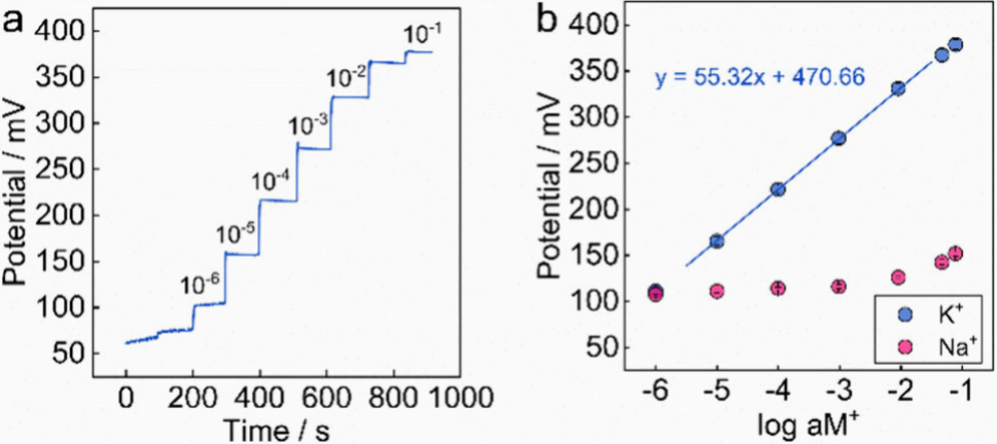
(a) Time trace of the potential signal at different K^+^ concentrations, (b) calibration cure and Na^+^ selectivity of the K^+^-selective electrode with molecularly thin PVC layer.

As a control, the K^+^-selective electrodes without any PVC substrate were prepared by directly applying the same cocktail onto the PEDOT layer, and the potentiometric performances were evaluated (Figure S5). The results show poor potentiometric response, with drifting signals and a non-Nernstian slope. The thin PVC layer is therefore essential for endowing the material with permselective sensing behavior. Despite these promising results, maintaining attractive sensing performance across all electrodes proved challenging, as sub-Nernstian responses were observed for some electrodes (Figure S4). A plausible explanation is the difficulty in controlling the uniformity of the sensing membrane, owing to the large difference in thickness of the PVC layer, compared to the PEDOT layer. This may make it difficult to avoid imperfections such as pin holes that would compromise electrochemical behavior. The response slope is 55.3 ± 0.5 mV from repeated calibrations.

SC-ISEs overlaid with drop-cast ion-selective membranes are known to exhibit excellent ion response properties and stability. To evaluate the resulting potential stability and reproducibility, an additional thick PVC membrane was solvent cast on top of the covalently attached thin PVC layer. As shown in Figure S6, the performance of the electrodes was significantly improved with the addition of a thick PVC membrane, with all electrodes exhibiting Nernstian responses. Moreover, the standard deviation of the standard potential (*E* ^0^) for these electrodes was remarkably reduced to 1.60 mV. This enhancement in *E* ^0^ reproducibility is likely attributed to the presence of the covalently attached PVC layer between the conducting polymer and the additional PVC membrane, which provides a well-defined interface and minimizes *E* ^0^ variability. To test this hypothesis, we fabricated three electrodes with thick PVC membranes but without the attached thin PVC layer (Figure S7, blue scatters). These electrodes exhibited much larger potential variations, with an *E* ^0^ standard deviation of 9.8 mV.

The sodium ion selectivity and time stability of the electrodes were evaluated (Figure S8). The Na^+^-logarithmic selectivity coefficient was log *K*_*I,j*_^pot^ = −4.05, comparable to that of classic solid-contact electrodes.^20^ Unfortunately, the electrodes exhibited limited stability with time, exhibiting a drop (14.73 mV) in *E* ^0^ within the first 2 days, followed by a slow drift over a two-week period (1.8 mV per day, see Figure S8b). This instability is hypothesized to result from water penetration through the additional PVC membrane, but further experiments are required to confirm and address this. The Nernstian slopes were maintained during a two-week measurement period, which confirms that the instability is caused by interfacial changes away from the membrane–sample interface.

In conclusion, we formed a controlled PVC membrane through CuAAC click chemistry. After doping with ionophores and ion exchangers, this ultrathin PVC membrane exhibited a near-Nernstian response with a logarithmic selectivity coefficient over Na^+^ of log *K*_*K*,Na_^pot^ = −4.10. The introduction of an additional solvent-cast PVC layer further improved analytical performance, reducing the standard deviation of the standard potential (*E* ^0^) to just 1.60 mV. The attached thin PVC film with controlled thickness down to the nanoscale offers a promising pathway for the development of highly stable, calibration-free SC-ISEs suitable for applications in clinical diagnostics, environmental monitoring, and wearable sensing.

## Supplementary Material



## Notes

Additional experimental details including experimental sections, electropolymerization voltammogram, water contact angle of PEDOT-alkyne layer, equivalent electrical circuit, calculation of Debye length, and potentiometry results (PDF)
