# Supplementary material for: Covalent Attachment of Molecularly Thin PVC Membrane by Click Chemistry for Ionophore-Based Ion Sensors
Source: Anal Chem. 2025 Aug 11;97(33):17917–20. doi: 10.1021/acs.analchem.5c01986 (PMC12392254; doi:10.1021/acs.analchem.5c01986)
Supplement: Supplementary file 1 [file ac5c01986_si_001.pdf]

Covalent Attachment of Molecularly Thin PVC Membrane by Click Chemistry for Ionophore-Based Ion Sensors

Yupu Zhang,[a] Tara Forrest,[a] Yaotian Wu,[a] Plinio Maroni [a] and Eric Bakker *[a]

Department of Inorganic and Analytical Chemistry, University of Geneva, Quai Ernest-Ansermet 30, CH-1211, Geneva, Switzerland

* Email: Eric.Bakker@unige.ch

Table of Content

Experiments..........S2

Figure S1..........S4

Figure S2..........S4

Figure S3..........S5

Figure S4..........S5

Figure S5..........S6

Figure S6..........S6

Figure S7..........S7

Figure S8..........S7

Debye length calculation in the plasticized PVC membrane..........S8

EXPERIMENTAL SECTION

Reagents. 2-Pent-4-ynyl-2,3-dihydro-thieno[3,4-b][1,4]dioxine (EDOT-alkyne) and PVC-N_3 were gifts from Selectoprobe (selectoprobe.com). Acetonitrile (ACN), tetrahydrofuran (THF), N,N-Dimethylformamide (DMF), tetrakis(acetonitrile)copper(I) hexafluorophosphate, tetrabutylammonium perchlorate ($TBAClO_4$), potassium chloride (KCl), sodium chloride (NaCl), valinomycin, sodium tetrakis[3,5-bis(tri-fluoromethyl)phenyl]borate (NaTFPB), di(2-ethylhexyl) sebacate (DOS), and tetrakis(acetonitril)kupfer(I)-hexafluorphosphat were purchased from Sigma-Aldrich. All solutions were prepared using deionized water (>18.2 MΩ·cm specific resistance).

Instrumentation. Potentiometric measurements were performed with a high impedance input 16-channel EMF monitor (Lawson Laboratories, Inc., Malvern, PA), using a double-junction Ag/AgCl/ 3 M KCl/ 1 M LiOAc electrode (Metrohm, Switzerland) as the reference electrode. An Autolab (PGSTAT 302 N, Metrohm) was used to perform the electropolymerization and impedance experiment, controlled by NOVA 2.1 software. Electrochemical quartz crystal microbalance (QCM, QSense Analyzer, Boilin Scientific) was used to monitor the mass difference during electropolymerization and click reaction. Water contact angle images were acquired with a Canon EOS 5D Mark III camera, using Image J software for image analysis.

Deposition of the PEDOT-alkyne layer. Glassy carbon (GC) electrodes (⌀ 3 ± 0.1 mm, Metrohm) were polished using different sizes of diamond spray (6, 3, 1, and 0.25 μm, respectively) before use. The polymerization solution for generating a clickable PEDOT layer was prepared by dissolving EDOT-alkyne (0.01 M) and $TBAClO_4$ (0.1 M) in acetonitrile. [1] The PEDOT-alkyne film was polymerized on the GC electrode by applying two voltammetric cycles (Figure S1, Applied potential: from -1.05 to 1.55 V at 50 mV s^{-1}).

Ion-selective electrode preparation. The electrodes with the PEDOT-alkyne layer were immersed into a PVC-N_3 solution (1 mg mL^{-1} in DMF), and a catalytic amount of tetrakis(acetonitrile)copper(I) hexafluorophosphate was added to initiate the reaction. The reaction was left overnight at room temperature, after which time the electrodes were rinsed with DMF and THF respectively. Water contact angle experiment, cyclic voltammetry, and impedimetric measurements were performed to evaluate the reaction completion. K^+-selective cocktails for molecularly-thin membrane electrode (133 mg of DOS, 0.89 mg of NaTFPB, 2.22 mg of valinomycin, 2 mL THF) and thick membrane electrode (133 mg of DOS, 67 mg PVC-N_3, 0.89 mg of NaTFPB, 2.22 mg of valinomycin, 2 mL THF) were prepared. 10 μL of the cocktail were dropped on top of the electrodes, allowing them to dry overnight. The prepared electrodes were conditioned in 1 mM KCl (K^+-ISEs) before evaluation.

Mass changes monitored by QCM. The mass changes during electropolymerization and the click reaction were monitored by electrochemical quartz crystal microbalance (QCM). A home-made cell was used for surface modification that consisted of two parts: one part is commercially available (QSense QEM-401) and connects to the QCM for signal transmission, and the other part features a round opening above the QCM sensor to facilitate surface modification by electrochemistry. A three-electrode system was used for electropolymerization, using a platinum reference electrode, a carbon counter electrode, and a gold QCM-D chip (Renlux Crystal, Shenzhen) working electrode. 300 μL of 0.01 M

EDOT-alkyne solution was added into the opening of the cell, and EQCM measurement was started before applying the potential controlled by the Autolab potentiostat, scanning from -1.05 to 1.55 V at a scan rate of 50 mV s^{-1} for 2 cycles. The gold substrate was then rinsed with ACN, THF, and DMF respectively, followed by the addition of 300 μL of PVC-N_3 solution containing catalyst. The measurements were stopped after no more mass difference were observed, about 100 min.

Layer thickness characterization using ellipsometry. The thickness of PEDOT-alkyne layer was determined in the air using ellipsometry (Multiskop, Optrel) with a glass with Au substrate. The angle of incidence used was 73 °, and the laser wavelength used was 632.8 nm. After electropolymerized PEDOT-alkyne, we performed the ellipsometry by setting the layer models (layer 0: air, layer 1: PEDOT-alkyne, layer 2: gold, and layer 3: glass) to measure and obtain Ψ and Δ data, which could be further fitted to obtain the layer thickness information. The real parts of the refractive indices applied were 1.00, 1.70, 0.18, and 1.51 for air, PEDOT-alkyne, gold, and glass, respectively, and the imaginary part of the refractive index for gold was -3.43.

Electrochemical impedance spectroscopy and contact angle measurements. Impedance measurements were performed with a three-electrode cell, using a double-junction Ag/AgCl reference electrode and a platinum counter electrode. Measurements were performed before and after the click reaction, with a frequency range of 0.01 Hz to 10^5 Hz at an open circuit potential of 0.22 V. The electrodes were tested in 0.1 M KCl, 0.01 M $K_3[Fe(CN)_6]/K_4[Fe(CN)_6]$ solution. For the water contact angle experiment, 1 μL of deionized water was pipetted on the electrode surface. A Canon EOS 5D Mark III camera was used to record the images.

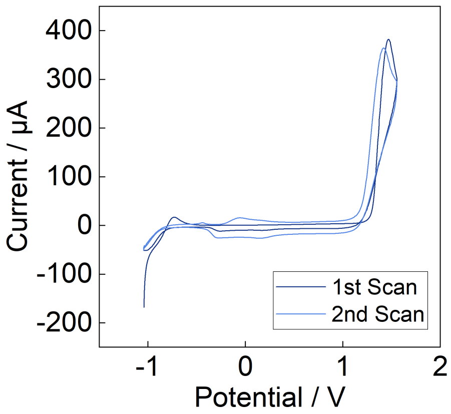

Figure S1. Voltammogram for PEDOT-alkyne electropolymerization. (Potential: -1.05-1.55 V, scan rate: 50 mV/s)

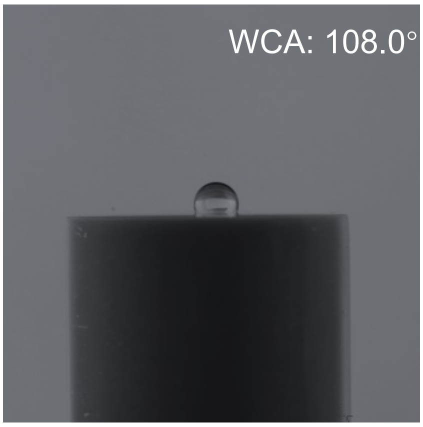

Figure S2. Water contact angle of PEDOT-alkyne layer on GC electrode (before click reaction).

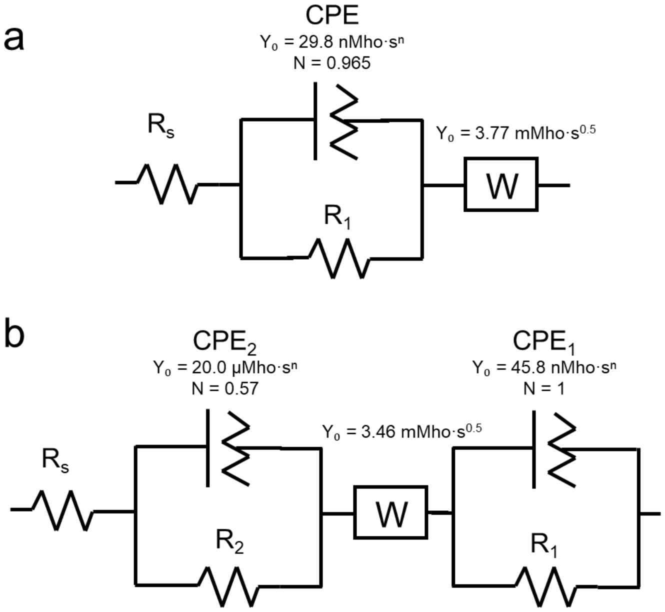

Figure S3. Equivalent electrical circuit for the GC electrode (a) with PEDOT-alkyne layer, (b) molecularly thin PVC layer attached on PEDOT. R_s, solution resistance; CPE: constant phase element.

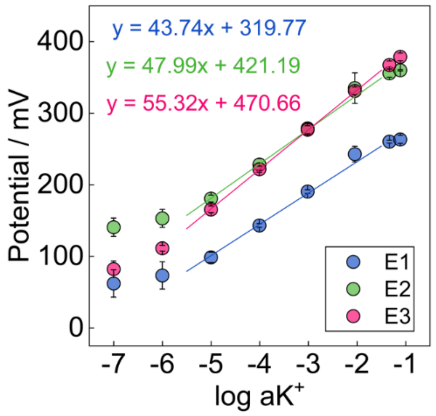

Figure S4. Calibration curves of the K^+-selective electrode with molecularly thin PVC layer.

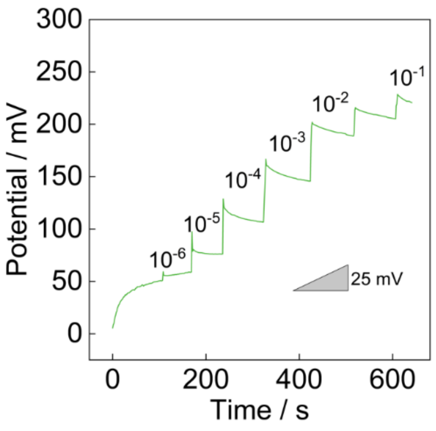

Figure S5. Time trace of the potential signal at different K^+ concentrations without PVC substrate.

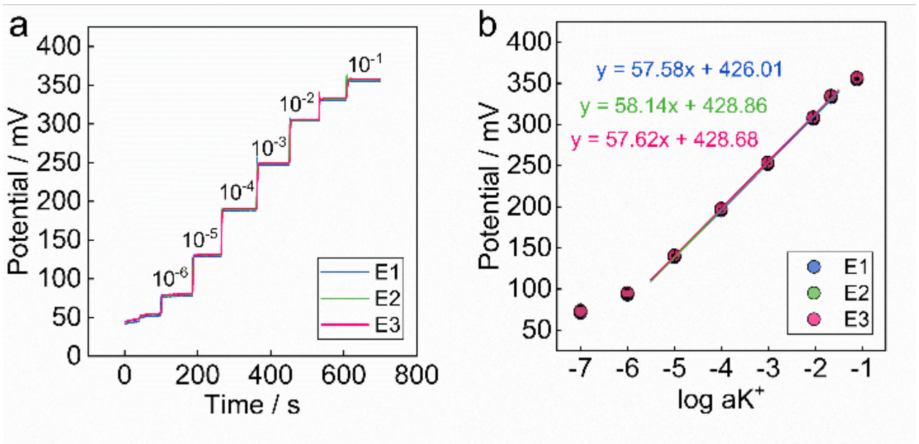

Figure S6. (a) Time traces of the potential signal at different K^+ concentrations, (b) calibration cures of the K^+-selective electrode with an additional PVC layer.

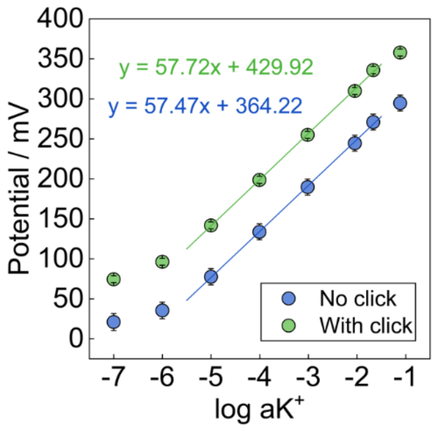

Figure S7. Comparison of E^0 reproducibility with overcoated membrane, with and without molecularly thin PVC layer on the ion to electron transducer.

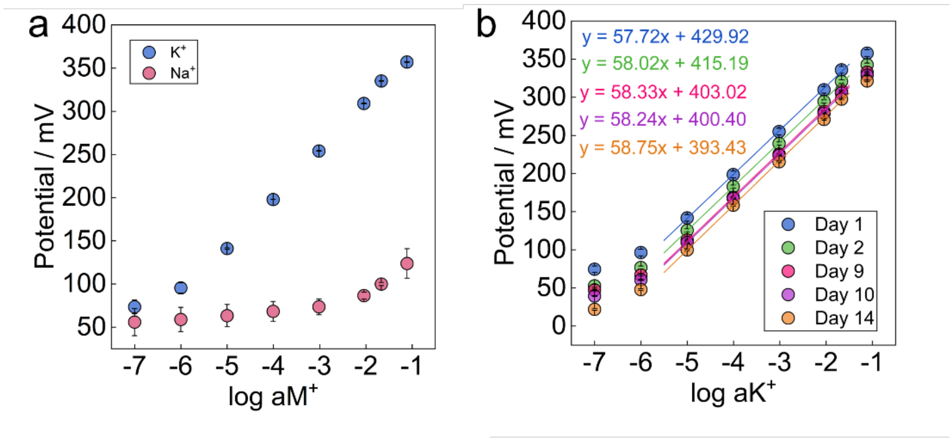

Figure S8. (a) K^+ response and Na^+ selectivity, (b) time stability of the K^+-selective electrode with an additional PVC layer.

Debye length in the plasticized PVC membrane

Based on the Debye–Hückel theory, the Debye length in our plasticized PVC membrane can be calculated using Equation S1.

$$\frac{1}{k} = \sqrt{\frac{\varepsilon_r \varepsilon_0 k_B T}{2 N_A e^2 I}} \qquad \text{(S1)}$$

where I is the ionic strength of the electrolyte, ε_0 is the permittivity of free space (8.854×10^{-12} F/m), ε_r is the dielectric constant, k_B is the Boltzmann constant (1.381×10^{-23} J/K), T is the absolute temperature in kelvins (298 K), N_A is the Avogadro's number (6.022×10^{23} mol^{-1}), and e is the elementary charge (1.602×10^{-19} C).

Calculation of ionic strength in the membrane.

In the cocktail used for molecularly-thin membrane electrode (133 mg of DOS, 0.89 mg of NaTFPB, 2.22 mg of valinomycin, 2 mL THF), the volume of DOS is 0.1452 mL, and the amount of NaTFPB added corresponds to 0.001 mmol. The concentration of NaTFPB in the membrane is calculated as

$$C = \frac{n}{V} = \frac{0.001\ mmol}{0.1452\ mL} = 6.89\ mM$$

After conditioning, the Na^+ is replaced by K^+, while the total ion concentration in the membrane remains unchanged. The ionic strength is calculated using the following equation

$$I = \frac{1}{2}\sum_{i=1}^{n} c_i z_i^2 = 6.89\ mM$$

Calculation of the dielectric constant in the membrane.

The thin membrane consists of both PVC (ε_r=3) and DOS (ε_r=4.5). The area of the glassy carbon (GC) electrode surface is 0.071 cm^2 (⌀ 3 ± 0.1 mm). Based on quartz crystal microbalance (QCM) measurements, the PVC mass per unit area is 5.93 μg/cm^2, giving a total PVC mass on the GC surface of:

$$m_{PVC} = 5.93\frac{\mu g}{cm^2} \times 0.071 cm^2 = 0.42\ \mu g$$

The membrane cocktail contained 133 mg of DOS in 2 mL of THF. When 10 μL of this solution was drop-cast onto the electrode, the total DOS mass was:

$$m_{DOS_{tot}} = 133\ mg \times \frac{10\ \mu L}{2\ mL} = 0.665\ mg$$

This mass was distributed over the full electrode surface (⌀ 10 mm), while the GC detection area is smaller (⌀ 3 mm). Thus, the corrected DOS mass on the GC area is:

$$m_{DOS} = 0.665\ mg \times \frac{\pi \times 3\ mm^2}{\pi \times 10\ mm^2} = 0.060\ mg$$

Comparing the mass of PVC and DOS on the GC surface, indicates that DOS is the dominant component by mass. Therefore, the effective dielectric constant of the membrane is expected to be close to that of DOS, i.e., $\varepsilon_r \approx \varepsilon_{DOS} = 4.5$.

By inserting all parameter values into Equation S1, the Debye length was calculated to be approximately 0.9 nm. This value is smaller than those reported in other studies, [2] which is attributed

to the higher ionic strength in our membrane system (6.89 mM), whereas in other reports the ionic strength is typically below 1 mM.

Reference

(1) Bu, H. B.; Gotz, G.; Reinold, E.; Vogt, A.; Schmid, S.; Blanco, R.; Segura, J. L.; Bauerle, P. Click-functionalization of conducting poly(3,4-ethylenedioxythiophene) (PEDOT). *Chem. Commun.* (Camb) **2008**, 1320-1322. DOI: 10.1039/b718077b.

(2) Morf, W. E.; Simon, W. Cation-Response Mechanism of Neutral Carrier Based Ion-Selective Electrode Membranes. *Helv. Chim. Acta* **1986**, *69*, 1120-1131.
